# *BMI1* is associated with CSF amyloid-β and rates of cognitive decline in Alzheimer’s disease

**DOI:** 10.1186/s13195-021-00906-4

**Published:** 2021-10-05

**Authors:** Jun Pyo Kim, Bo-Hyun Kim, Paula J. Bice, Sang Won Seo, David A. Bennett, Andrew J. Saykin, Kwangsik Nho, for the Alzheimer’s Disease Neuroimaging Initiative

**Affiliations:** 1 Center for Neuroimaging, Radiology and Imaging Sciences, Indiana University School of Medicine, 355 W 16th St. Methodist hospital, GH 4101, Indianapolis, Indiana 46202 USA; 2 Medical Research Institute, Sungkyunkwan University School of Medicine, Seoul, South Korea; 3 Department of Biomedical Engineering, Hanyang University, Seoul, South Korea; 4 Department of Neurology, Samsung Medical Center, Sungkyunkwan University School of Medicine, Seoul, South Korea; 5 Rush Alzheimer’s Disease Center, Rush University Medical Center, Chicago, IL USA; 6 Indiana Alzheimer Disease Center, Indiana University School of Medicine, Indianapolis, Indiana USA; 7 Department of Medical and Molecular Genetics, Indiana University School of Medicine, Indianapolis, IN USA; 8 Center for Computational Biology and Bioinformatics, Indiana University School of Medicine, Indianapolis, Indiana USA

**Keywords:** Alzheimer’s disease, Neurogenetics, Amyloid, Cognition

## Abstract

**Background:**

Accumulating evidence suggests that *BMI1* confers protective effects against Alzheimer’s disease (AD). However, the mechanism remains elusive. Based on recent pathophysiological evidence, we sought for the first time to identify genetic variants in *BMI1* as associated with AD biomarkers, including amyloid-β.

**Methods:**

We used genetic, longitudinal cognition, and cerebrospinal fluid (CSF) biomarker data from participants in the Alzheimer’s Disease Neuroimaging Initiative (ADNI) cohort (*N* = 1565). First, we performed a gene-based association analysis of common single nucleotide polymorphisms (SNPs) (minor allele frequency (MAF) > 5%) located within ± 20 kb of the gene boundary of *BMI1*, an optimal width for including potential regulatory SNPs in the 5′ and 3′ untranslated regions (UTR) of *BMI1*, with CSF Aβ_1-42_ levels. Second, we performed cross-sectional and longitudinal association analyses of SNPs in *BMI1* with cognitive performance using linear and mixed-effects models. We replicated association of SNPs in *BMI1* with cognitive performance in an independent cohort (*N*=1084), Religious Orders Study and the Rush Memory and Aging Project (ROS/MAP).

**Results:**

Gene-based genetic association analysis showed that *BMI1* was significantly associated with CSF Aβ_1-42_ levels after adjusting for multiple testing using permutation (permutation-corrected *p* value=0.005). rs17415557 in *BMI1* showed the most significant association with CSF Aβ_1-42_ levels. Participants with minor alleles of rs17415557 have increased CSF Aβ_1-42_ levels compared to those with no minor alleles. Further analysis identified and replicated the minor allele of rs17415557 as being significantly associated with slower cognitive decline rates in AD.

**Conclusions:**

Our findings provide fundamental evidence that *BMI1* rs17415557 may serve as a protective mechanism related to AD pathogenesis, which supports the results of previous studies linking *BMI1* to protection against AD.

**Supplementary Information:**

The online version contains supplementary material available at 10.1186/s13195-021-00906-4.

## Introduction

The etiology of non-familial Alzheimer's disease (AD) remains unclear despite extensive research efforts. In terms of genetic risks, researchers have focused on multiple common genetic risk factors with low effect sizes [1]. Recent large-scale genome wide association studies (GWAS) have identified more than 20 AD susceptibility loci [2]. Although common genetic variants have relatively small individual impact, the overall effect of multiple genetic risks can significantly increase the likelihood of developing AD [3].

The *BMI1* gene encodes a 37kDa protein BMI1, a component of the polycomb repressive complex 1 (PRC1). BMI1 is involved in cell development, DNA damage response, cellular senescence regulation, stem cell renewal and differentiation, and oncogenesis [4]. In terms of aging, the reduction of *BMI1* expression in aging cells was reported in vitro and in vivo studies [5, 6]. This might be related to one of the functions of *BMI1*, repressing cellular senescence [7]. Furthermore, recent studies have shown that *BMI1* expression is reduced in AD brains but not in other types of dementia, such as frontotemporal dementia or dementia with Lewy bodies [8]. In line with that, *BMI1* knock-out induced pluripotent stem cell (iPSC)-derived neurons induced pathologic characteristics of AD [8], and a mouse model study showed increased amyloid plaque, total Tau, and p-Tau levels in aged *Bmi1*-haplodeficient (*Bmi1*+/-) mice [9].

Although the association between *BMI1* and AD in terms of gene expression and protein concentration has been reported [8], the effect of single nucleotide polymorphisms (SNPs) in the *BMI1* gene in AD has not been studied. In view of recent pathophysiological evidence, we sought for the first time to investigate whether genetic variation in *BMI1* is associated with a core AD biomarker and cognitive decline. Here, we report a genetic association analysis of SNPs in *BMI1* with CSF Aβ_1-42_ levels and longitudinal cognitive performance in the Alzheimer’s Disease Neuroimaging Initiative (ADNI) cohort. We replicated association of SNPs in *BMI1* with cognition performance in an independent Religious Orders Study and the Rush Memory and Aging Project (ROS/MAP) cohort.

## Materials and methods

### Subjects

Data used in the study were obtained from the Alzheimer’s Disease Neuroimaging Initiative (ADNI), a publicly available database (https://adni.loni.usc.edu) [10, 11]. A total of 1565 participants had genetic data. Of those participants, we used 1157 participants with cerebrospinal fluid (CSF) amyloid-β1-42 (Aβ_1-42_) levels and 1495 participants with longitudinal cognitive performance data (Table 1, Supplementary Figure 1).

**Table 1:** Participants characteristics

	ADNI		ROS/MAP
	CSF dataset	ADAS-cog 13 dataset	
Number of subjects, *n*	1157	1495	1084
Age, years (mean (SD))	73.0 (7.3)	73.5 (7.2)	80.5 (6.8)
Male sex, *n* (%)	509 (44.0)	850 (56.9)	361 (33.3)
Education, years (mean (SD))	16.1 (2.8)	16.0 (2.8)	16.43 (3.6)
APOE ε4 carrier, *n* (%)	544 (47.0)	709 (47.4)	284 (26.2)
Follow-up duration, years (mean (SD))	–	4.3 (3.0)	7.5 (4.6)
Diagnosis, *n* (%)^a^			
CN	337 (29.1)	445 (29.8)	683 (63.0)
MCI	594 (51.3)	763 (51.0)	312 (28.8)
Dementia	226 (19.5)	287 (19.2)	89 (8.2)
CSF Aβ_42_, pg/mL (mean (SD))	1038.8(600.9)	–	–
ADAS-cog 13, (mean (SD))	–	17.0 (9.5)	–
Global cognition composite score, (mean (SD))	–	–	-0.125 (0.631)
Amyloid positivity, *n* (positive/negative/missing)	843/314/0	882/359/254	–

Data are presented as mean (standard deviation) for continuous variables and *n* (%) for categorical variables

*ADNI* Alzheimer’s Disease Neuroimaging Initiatives, *CSF* cerebrospinal fluid, *ADAS* Alzheimer’s disease assessment scale, *ROS* Religious Orders Study, *MAP* Memory and Aging Project, *Aβ* amyloid beta, *CN* cognitively normal, *MCI* mild cognitive impairment

^a^For CSF data, the diagnosis information when the CSF was drawn was used. For ADAS-cog 13, the initial diagnosis was used

Additionally, we used an independent dataset to validate ADNI findings from association analysis between genotype and cognitive function. The dataset is from two large cohorts maintained by investigators at the Rush Alzheimer’s Disease Center: the Religious Orders Study (ROS) and the Rush Memory and Aging Project (MAP) [12]. A total of 1084 participants had both genetic and longitudinal cognitive performance data.

### Genotyping

The ADNI participants were genotyped using several Illumina genotyping platforms. Quality control (QC) procedures for participants and SNPs were performed as described previously [13]. After QC procedures, we selected only non-Hispanic participants of European ancestry and imputed un-genotyped SNPs separately in each platform using Markov Chain Haplotyping with the Haplotype Reference Consortium data as a reference panel [14]. The ROS/MAP whole genome sequencing libraries were prepared using the KAPA Hyper Library Preparation Kit in accordance with the manufacturer’s instructions and sequenced on an Illumina HiSeq X sequencer using pair-end read chemistry and read lengths of 150bp. The paired-end 150bp reads were aligned to the NCBI reference human genome (GRCh37) using the Burrows-Wheeler Aligner (BWA-MEM) [14, 15]. Local alignment was performed around indels to identify putative insertions or deletions in the region using the GATK (version 3.5) indel realignment tool. Base quality score recalibration was performed using the GATK BQSR. Variant calling and QC procedures have been described elsewhere [16]. Briefly, GATK HaplotypeCaller and GenotypeGVCFs modules were used to generate individual genotype calls in genomic VCF and VCF format. Following variant calling, the variant quality recalibration step in the GATK pipeline was used to empirically calibrate high quality variants. Variant-level QC was performed using PLINK, which includes checking genotype concordance using previous GWAS data, excluding variants with excess and/or systematic genotype missingness, examining departure from Hardy-Weinberg Equilibrium, and identifying Mendelian inconsistencies among related individuals.

### CSF biomarkers

In ADNI, CSF Aβ_1-42_ and phosphorylated Tau (p-Tau) levels were measured by validated and highly automated Roche Elecsys electrochemiluminescence immunoassays (Roche Diagnostics, Mannheim, Germany) [17].

### Amyloid positivity

Amyloid positivity was determined using CSF Aβ_1-42_ levels and ^18^F-Florbetapir positron emission tomography (PET) standardized uptake value ratios (SUVRs). In terms of CSF Aβ_1-42,_ the provisional cut-point of 1073 pg/ml was used and 1.11 was used as the cut-point for ^18^F-Florbetapir PET SUVR. Participants who tested positive at least once during the follow up were labeled as amyloid positive.

### Cognitive performance measures

As a cognitive performance measure for the ADNI participants, we used the AD Assessment Scale-cognitive subscale 13 (ADAS-cog13) [18], which includes 13 items (Word Recall, Naming Objects and Fingers, Commands, Constructional Praxis, Ideational Praxis, Orientation, Word Recognition, Language, Comprehension of Spoken Language, Word Finding Difficulty, Remembering Test Instructions, Delayed Word Recall, Number Cancellation or Maze Task) related to fundamental cognitive functions. The ROS/MAP participants underwent cognitive assessment using a battery of 21 cognitive performance tests. Nineteen of these tests across a range of cognitive abilities including 7 episodic memory tests (Word List Memory, Word List Recall, Word List Recognition, immediate and delayed recall of the East Boston Story and Story A from Logical Memory), 3 semantic memory tests (15-item Boston Naming Test, verbal fluency, 15-item word reading test), 3 working memory tests (Digit Span Forward, Digit Span Backward, Digit Ordering), 2 perceptual orientation tests (Line Orientation, 16-item progressive matrices), and 4 perceptual speed tests (Symbol Digits Modality-oral, Number Comparison, Stroop Color Naming, Stroop Word Naming) were used to construct a global composite measure of cognitive function. Further, information on this composite measure is published elsewhere [19, 20].

### Statistical analysis

For gene-based association analysis, we selected common SNPs (MAF >5%) located within ± 20 kb of upstream and downstream regions of the *BMI1* gene. We chose the 20 kb window, which provides an optimal width for including potential regulatory SNPs in the 5′ and 3′ untranslated regions (UTR) of *BMI1*, while controlling for false SNP-to-gene mappings due to larger windows. The gene-based association analysis with additive genetic models was performed using a set-based test in PLINK. Permutation (20,000 permutations) was used to adjust for multiple testing, which calculated an empirical *p*-value to determine the statistical significance of all SNPs in *BMI1* jointly. For CSF Aβ_1-42_ levels, age, sex, and *APOE* ε4 carrier status were used as covariates. Furthermore, we performed association analysis of the SNP showing the most significant association with cognitive performance. We used a linear regression model with age, sex, and educational attainment as covariates. Longitudinal association analysis of the SNP with rates of cognitive decline was performed using a linear mixed-effects model under a missing at random hypothesis. The SNP genotype, time, the interaction term (SNP * time), age, sex, and educational attainment were treated as main effects. Random intercepts and slopes for time were used to accommodate individual variation. Because most (≃ 95%) ADNI participants had a follow-up period shorter than 10 years, we used data points up to ten years from baseline to ensure the robustness of our results. For the same reason, we included data points up to 17 years of follow-up for ROS/MAP. We performed sex- and *APOE* ε4 carrier status- and β-amyloid positivity-stratified analysis. In addition to the stratified analyses, we investigated the interaction between the SNP in *BMI1* and grouping variables (sex, *APOE* ε4 carrier status, β-amyloid positivity). Because CSF Aβ_1-42_ levels and ADAS-cog13 scores showed a skewed distribution, we normalized the data using log transformation and square root transformation, respectively.

## Results

**Fig. 1:**
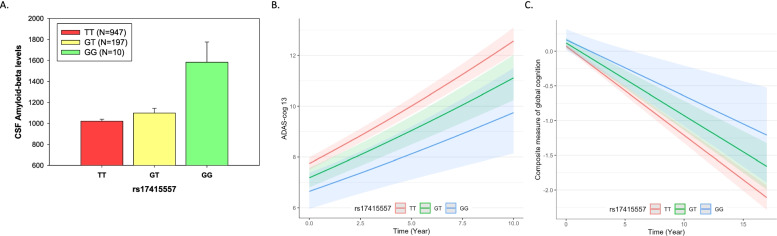
Association of *BMI* rs17415557 with CSF Aβ_1-42_ levels and rates of cognitive decline. **A** Participants with minor alleles (G) of rs17415557 have higher CSF Aβ_1-42_ levels, compared to those with no minor alleles (*β* (SE) = 0.05(0.02), *p* = 0.001). Participants with minor alleles of *BMI1* rs17415557 had slower cognitive decline over time compared to those with no minor alleles; for **B**, ADAS-cog13 (a higher score indicating poor cognitive function) in ADNI, *β* (SE) = − 0.035 (0.016) and *p* = 0.024; for **C**, a global composite measure of cognitive function (a lower score indicating poor cognitive function) in ROS/MAP, *β* (SE) = 0.024 (0.010) and *p* = 0.020

We analyzed eight common SNPs (MAF > 5%) located within ± 20 kb of the gene boundary of *BMI1* from HRC-based imputed ADNI GWAS data. Gene-based association analysis showed that *BMI1* was significantly associated with CSF Aβ_1-42_ levels (permutation-corrected *p* = 0.005). The significance of associations, genomic locations, and linkage disequilibrium information between the eight SNPs are shown in Supplementary Figure 2. Among the eight SNPs, rs17415557 showed the most significant association with CSF Aβ_1-42_ levels (β (SE) = 0.116 (0.035), *p* = 0.001). This SNP was highly correlated with rs72814833 (*R*^2^ = 0.99), a 697 base pair upstream (5′) variant of *BMI1*, which was also significantly associated with CSF Aβ_1-42_ levels (*β* (SE) = 0.116 (0.036), *p* = 0.001). Participants with minor alleles (G) of rs17415557 have higher CSF Aβ_1-42_ levels, compared to those with no minor alleles (Fig. 1A). Stratified analysis by sex and amyloid positivity showed that this association was pronounced in males (male: *β* (SE) = 0.151 (0.047), *p* = 0.001; female: *β* (SE) = 0.075 (0.052), *p* = 0.152) and in amyloid-positive participants (amyloid-negative: *β* (SE) = 0.052 (0.035), *p* = 0.131; amyloid-positive: *β* (SE) = 0.099 (0.034), *p* = 0.004) (Table 2), although no significant interactions were found (*p* = 0.276 for sex * rs17415557, *p* = 0.473 for amyloid positivity* rs17415557). The association was significant in both *APOE* ε4 carrier status groups when stratified. For CSF p-Tau, a tau biomarker for AD, we did not find any significant associations between rs17415557 and CSF p-Tau levels (*β* (SE) = − 0.020 (0.032), *p* = 0.521).

**Table 2:** Association of *BMI1* rs17415557 with CSF Aβ_42_ and cognitive function

	*N*	Cross-sectional^a^			Longitudinal^b^		
		*β*	SE	*p* value	*β*	SE	*p* value
ADNI dataset							
CSF Aβ_42_							
All subjects	1157	0.116	0.035	**0.001**			
Male	509	0.151	0.047	**0.001**			
Female	648	0.075	0.052	0.152			
ε4 non-carrier	613	0.109	0.049	**0.028**			
ε4 carrier	544	0.120	0.049	**0.014**			
Amyloid (−)	314	0.052	0.035	0.131			
Amyloid (+)	843	0.099	0.034	**0.004**			
ADAS-cog 13							
All subjects	1495	− 0.194	0.070	**0.006**	− 0.035	0.016	**0.024**
Male	850	− 0.210	0.086	**0.014**	− 0.021	0.019	0.277
Female	645	− 0.166	0.117	0.158	− 0.051	0.025	**0.044**
ε4 non-carrier	786	− 0.147	0.089	0.097	− 0.027	0.016	0.091
ε4 carrier	709	− 0.185	0.104	0.076	− 0.029	0.027	0.286
Amyloid (−)	359	− 0.081	0.099	0.413	− 0.020	0.016	0.219
Amyloid (+)	882	− 0.255	0.095	**0.008**	− 0.041	0.021	**0.049**
ROS/MAP dataset							
Global cognition score							
All subjects	1084	0.065	0.041	0.116	0.024	0.010	**0.020**
Male	361	0.029	0.075	0.696	0.007	0.017	0.670
Female	723	0.089	0.048	0.067	0.032	0.013	**0.012**
ε4 non-carrier	800	0.071	0.044	0.107	0.021	0.010	**0.042**
ε4 carrier	284	− 0.016	0.095	0.865	0.023	0.026	0.383

*ADNI* Alzheimer’s Disease Neuroimaging Initiatives, *CSF* cerebrospinal fluid, *ADAS* Alzheimer’s disease assessment scale, *ROS* Religious Orders Study, *MAP* Memory and Aging Project, *Aβ* amyloid beta

^a^Multiple linear models accounting for age, sex, *APOE* genotype, and educational attainment were tested. Regression statistics of the main effect “rs17415557” in each model are shown

^b^Linear mixed-effects models accounting for age, sex, *APOE* genotype, and educational attainment were tested. Regression statistics of the interaction term “rs17415557 * time” in each model are shown

For cognition performance at baseline, participants with minor alleles of rs17415557 showed higher ADAS-cog 13 scores in ADNI (*β* (SE) = 0.194 (0.070), *p* = 0.006). This association was pronounced in males (male: *β* (SE) = − 0.210 (0.086), *p* = 0.014; female: *β* (SE) = − 0.166 (0.117), *p* = 0.158) and in amyloid-positive participants (amyloid-negative: *β* (SE) = − 0.081 (0.099), *p* = 0.413; amyloid-positive: *β* (SE) = − 0.255 (0.095), *p* = 0.008). However, none of these cross-sectional association results of cognitive performance was replicated in ROS/MAP.

In order to investigate the effect of rs17415557 on rates of cognitive decline, we performed a longitudinal analysis of cognitive performance in two independent cohorts. The longitudinal analysis identified and replicated the significant association of rs17415557 with rates of cognitive decline (Fig. 1B, C). Participants with minor alleles of *BMI1* rs17415557 had slower cognitive decline over time compared to those with no minor alleles, for ADAS-cog13 (a higher score indicating poor cognitive function) in ADNI, *β* (SE) = − 0.035 (0.016) and *p* = 0.024; for a global composite measure of cognitive function (a lower score indicating poor cognitive function) in ROS/MAP, *β* (SE) = 0.024 (0.010) and *p* = 0.020. The sex-stratified analysis showed that the impact of rs17415557 on rates of cognitive decline was stronger in females in both cohorts (for ADNI, male: *β* (SE) = − 0.021 (0.019), *p* = 0.277; female: *β* (SE) = − 0.051(0.025), *p* = 0.044; for ROS/MAP, male: *β* (SE) = 0.007 (0.017), *p* = 0.670; female: *β* (SE) = 0.032 (0.013), *p* = 0.012) (Table 2). However, the interaction between sex and the rate of cognitive decline (sex * rs17415557 * time) was not significant (*p*= 0.539).

We identified rs72814833, which is closely correlated with rs17415557 (*R*^2^ = 0.99) and a 697 base pair upstream (5′) variant of *BMI1*, as significantly associated with CSF Aβ_1-42_ levels, cognitive performance at baseline, and rates of cognitive decline, which was replicated in ROSMAP, as expected due to the strong correlation between rs72814833 and rs17415557 (Supplementary table 1).

## Discussion

Here, we investigated the influence of genetic variants in *BMI1* on CSF Aβ_1-42_ levels and rates of cognitive decline. Our gene-based association analysis showed that *BMI1* was significantly associated with CSF Aβ_1-42_ levels. *BMI1* rs17415557 with the most significant association with CSF Aβ_1-42_ levels was also significantly associated with rates of cognitive decline, which was replicated in an independent cohort. Notably, the T to G substitution of rs17415557 was associated with higher CSF Aβ_1-42_ levels and slower cognitive decline over time. In a recent large-scale GWAS from the International Genomics of Alzheimer’s Project [1], the major allele (T) of rs17415557 was nominally associated with AD (*β* (SE) = 0.071 (0.035), *p* = 0.045). These results imply that the minor allele (G) of rs17415557 may have a protective effect against AD.

Our first major finding was that *BMI1* rs17415557 is associated with CSF Aβ levels. Interestingly, these findings were prominent within amyloid-positive subjects. This result is in line with a previous study where *BMI1* gene expression levels were decreased only in AD and not in other dementias [8], since amyloid positivity is a hallmark of AD. One possible explanation for the relationship between *BMI1* and AD suggested in a previous study is that *BMI1* deficiency leads to increased p53 and GSK3β levels, which can impair proteasome function [8]. However, the role and molecular mechanism that *BMI1* rs17415557 polymorphism may specifically play in the pathogenesis of AD warrants further investigation. CSF phosphorylated tau (pTau) levels were not significantly associated with *BMI1* rs17415557, although the direction of association was consistent with that of CSF Aβ levels. Previous studies showed significant associations between *BMI1* and pTau levels based on gene or transcript levels. Further investigations are needed to validate our finding for the association of CSF pTau levels with *BMI1* rs17415557.

Another major finding was that the minor allele (G) of rs17415557 had a significant protective effect on cognitive decline over time. AD is a chronic progressive disorder showing substantial individual variation in the time-course of cognitive decline [21], making it crucial to predict the clinical trajectory [22]. rs17415557 appears to contribute to this clinical course variability. Notably, the protective effect of the G allele on cognitive decline is most prominent among females. It is not unusual for a SNP to have a differential effect between sexes. The *APOE* ε4 allele is also differentially associated with cognitive decline in males and females, particularly having a more significant impact on females [23].

*BMI1* rs72814833, which is a 697 base pair upstream (5′) variant of *BMI1* and highly correlated with rs17415557, is also significantly associated with CSF Aβ_1-42_ levels and rates of cognitive decline. *BMI1* rs72814833 is known to bind with Egr1 protein as determined by the HaploReg v4.1 online tool (https://pubs.broadinstitute.org/mammals/haploreg/haploreg.php) (Supplementary Table 2). Egr1 is a member of a zinc-finger transcription factor family, and a previous mouse model study showed that Egr1 −/− hematopoietic stem cells exhibited significantly elevated levels of *BMI1* expression [24]. Although speculative, polymorphisms in the protein binding site might have influenced the action of Egr1, which could have contributed to our results. Our analysis for 3D chromatin interactions near *BMI1* rs17415557 showed that several distant genes, including *ARMC3*, *DNAJC1*, and *SKIDA1*, also interacted with regions near rs17415557 at 3D chromatin, and their expression might also be regulated by rs17415557.

## Limitations

A few limitations of this study should be noted. First, replication studies with independent larger samples are needed to confirm the association of *BMI1* with CSF Aβ_1-42_ levels. Second, the mechanism by which the identified SNPs in *BMI1* affect the *BMI1* gene and hence Aβ level is unknown. Besides the protein-binding properties of the identified SNPs found on a public database, further functional studies are needed to determine the specific biochemical mechanism. Third, for cognitive performance, ADAS-cog13 was used in ADNI, whereas a different global composite measure of cognitive function was used in ROS/MAP. Nevertheless, it is noteworthy that this is the first study to show that rs17415557 and rs72814833, genetic variants located in the upstream region of the *BMI1* gene, may play a protective role against AD.

## Conclusion

In conclusion, the findings of our study from two independent well-characterized cohorts provide fundamental evidence that *BMI1* and SNPs (rs17415557 and rs72814833) in *BMI1* are associated with CSF Aβ_1-42_, a hallmark biomarker of AD, and cognitive decline rates. These findings support results of previous studies linking *BMI1* to protection against AD [4, 5, 8]. Further studies, including animal models, are needed to investigate the molecular mechanisms underlying our findings.

## Supplementary Information

**Additional file 1: Supplementary Figure 1.** Flowchart for inclusion and exclusion of participants. ADNI = Alzheimer’s Disease Neuroimaging Initiative, GWAS = Genome-wide association study, CSF = Cerebrospinal Fluid, ADAS-cog = Alzheimer’s Disease Assessment Scale-cognitive subscale.

**Additional file 2: Supplementary Figure 2.** Visualization of genomic locations, associations with Aβ, and linkage disequilibrium of eight SNPs. (A) Association map of the eight SNPs within 20kb of *BMI1* gene (B) Linkage disequilibrium statistics (D’) between SNPs are shown.

**Additional file 3: Supplementary Table 1.** Association of *BMI1* rs72814833 with CSF Aβ42 and global cognitive function. Results of cross-sectional and longitudinal association analysis regarding *BMI1* rs72814833.

**Additional file 4: Supplementary Table 2.** Regulatory effects of the two SNPs of the *BMI1* gene (HaploReg, v4.1, update 05.11.2015). Annotation results of *BMI1* rs17415557 and rs72814833 from HaploReg v4.1web tool.

## Data Availability

The datasets generated and/or analyzed during the current study are available in the ADNI repository, (https://ida.loni.usc.edu/login.jsp?project=ADNI#) and AMP-AD Knowledge Portal (https://adknowledgeportal.synapse.org/).

## Abbreviations

AD: Alzheimer’s disease
CSF: Cerebrospinal fluid
ADNI: Alzheimer’s disease neuroimaging initiative
SNP: Single nucleotide polymorphism
ROS/MAP: Religious Orders Study and the Rush Memory and Aging Project
PRC1: Polycomb repressive complex 1
iPSC: Induced pluripotent stem cell
Aβ: Amyloid-β
NCBI: National Center for Biotechnology Information
QC: Quality control
PET: Positron emission tomography
SUVR: Standardized uptake value ratio
ADAS-cog13: Alzheimer’s Disease Assessment Scale-cognitive subscale 13
MAF: Minor allele frequency
SE: Standard error
