# Correction to: *BMI1* is associated with CSF amyloid-β and rates of cognitive decline in Alzheimer’s disease

**DOI:** 10.1186/s13195-022-00960-6

**Published:** 2022-01-20

**Authors:** Jun Pyo Kim, Bo-Hyun Kim, Paula J. Bice, Sang Won Seo, David A. Bennett, Andrew J. Saykin, Kwangsik Nho, for the Alzheimer’s Disease Neuroimaging Initiative

**Affiliations:** 1 Center for Neuroimaging, Radiology and Imaging Sciences, Indiana University School of Medicine, 355 W 16th St. Methodist hospital, GH 4101, Indianapolis, IN 46202 USA; 2 Medical Research Institute, Sungkyunkwan University School of Medicine, Seoul, South Korea; 3 Department of Biomedical Engineering, Hanyang University, Seoul, South Korea; 4 Department of Neurology, Samsung Medical Center, Sungkyunkwan University School of Medicine, Seoul, South Korea; 5 Rush Alzheimer’s Disease Center, Rush University Medical Center, Chicago, IL USA; 6 Indiana Alzheimer Disease Center, Indiana University School of Medicine, Indianapolis, IN USA; 7 Department of Medical and Molecular Genetics, Indiana University School of Medicine, Indianapolis, USA

**Correction to: Alzheimers Res Ther 13, 164 (2021)**

**https://doi.org/10.1186/s13195-021-00906-4**

Following the publication of the original article [1] the authors noticed that an 8 was inserted between CS and F of the word CSF in the article title. The correct article title should be:

“*BMI1* is associated with CSF amyloid-β and rates of cognitive decline in Alzheimer’s disease”

The original article [1] has been updated.

## References

[1] Kim JP, Kim BH, Bice PJ (2021). *BMI1* is associated with CSF amyloid-β and rates of cognitive decline in Alzheimer’s disease. Alzheimers Res Ther.

