# Supplementary figures and images for: BMI1 is associated with CSF amyloid-β and rates of cognitive decline in Alzheimer’s disease
Source: Alzheimers Res Ther. 2021 Oct 5;13:164. doi: 10.1186/s13195-021-00906-4 (PMC8493672; doi:10.1186/s13195-021-00906-4)

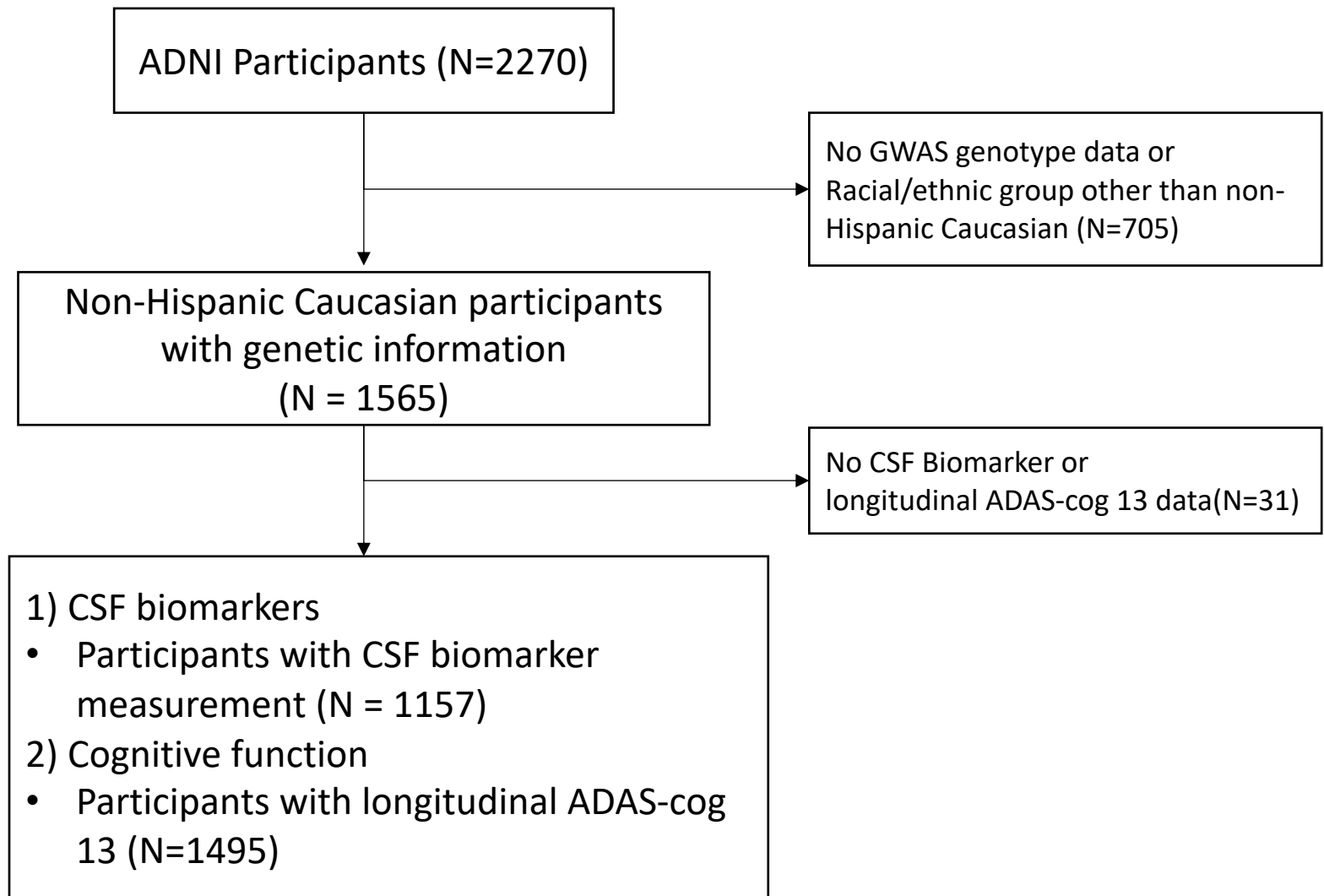

Supplement: Supplementary file 1 — Additional file 1: Supplementary Figure 1. Flowchart for inclusion and exclusion of participants. ADNI = Alzheimer’s Disease Neuroimaging Initiative, GWAS = Genome-wide association study, CSF = Cerebrospinal Fluid, ADAS-cog = Alzheimer’s Disease Assessment Scale-cognitive subscale. [file 13195_2021_906_MOESM1_ESM.pdf]

|| | | |

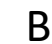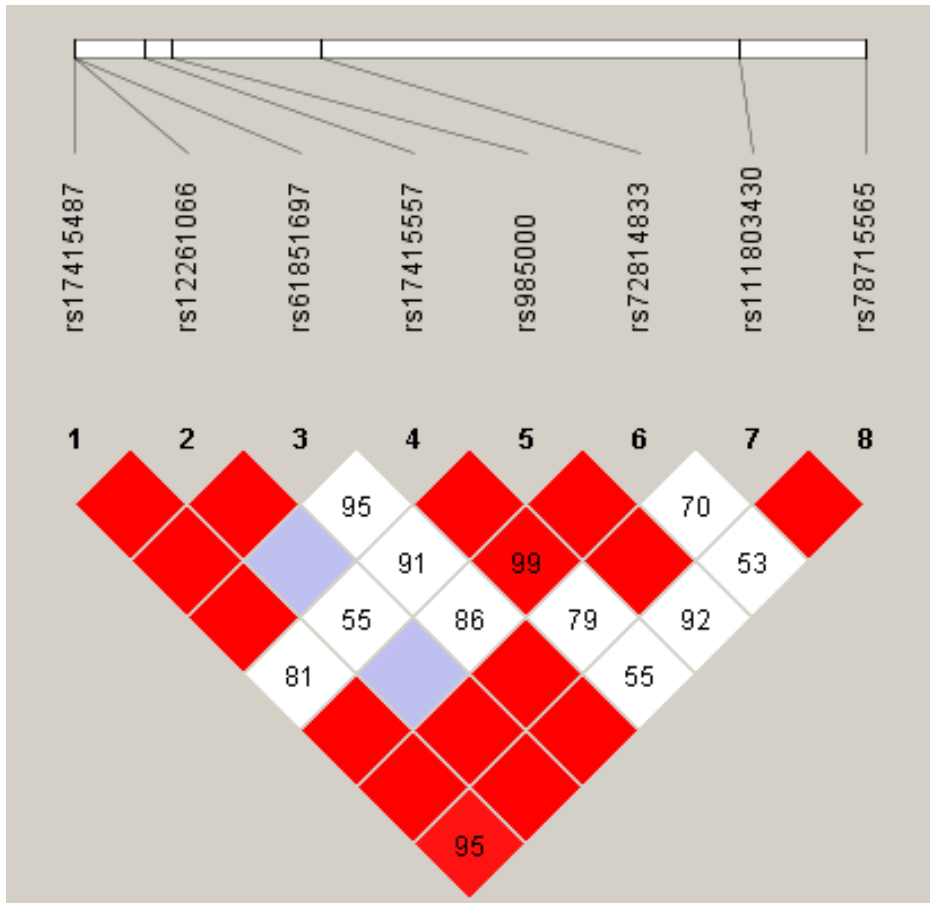

Supplement: Supplementary file 2 — Additional file 2: Supplementary Figure 2. Visualization of genomic locations, associations with Aβ, and linkage disequilibrium of eight SNPs. (A) Association map of the eight SNPs within 20kb of BMI1 gene (B) Linkage disequilibrium statistics (D’) between SNPs are shown. [file 13195_2021_906_MOESM2_ESM.pdf]
