# Supplementary material for: BMI1 is associated with CSF amyloid-β and rates of cognitive decline in Alzheimer’s disease
Source: Alzheimers Res Ther. 2021 Oct 5;13:164. doi: 10.1186/s13195-021-00906-4 (PMC8493672; doi:10.1186/s13195-021-00906-4)
Supplement: Supplementary file 3 — Additional file 3: Supplementary Table 1. Association of BMI1 rs72814833 with CSF Aβ42 and global cognitive function. Results of cross-sectional and longitudinal association analysis regarding BMI1 rs72814833. [file 13195_2021_906_MOESM3_ESM.docx]

Supplementary Table 1. Association of *BMI1* rs72814833 with CSF Aβ_42_ and global cognitive function

|  |  |  |  | Cross-sectional^†^ | | |  | Longitudinal^‡^ | | |
| --- | --- | --- | --- | --- | --- | --- | --- | --- | --- | --- |
|  |  |  | N | β | SE | p-value |  | β | SE | p-value |
| ADNI dataset | | | | | | | | | | |
|  | CSF Aβ_42_ |  |  |  |  |  |  |  |  |  |
|  | All subjects |  | 1157 | 0.116 | 0.036 | **0.001** |  |  |  |  |
|  | Male |  | 509 | 0.152 | 0.048 | **0.002** |  |  |  |  |
|  | Female |  | 648 | 0.073 | 0.053 | 0.171 |  |  |  |  |
|  | ε4 non-carrier |  | 613 | 0.104 | 0.050 | **0.038** |  |  |  |  |
|  | ε4 carrier |  | 544 | 0.124 | 0.050 | **0.013** |  |  |  |  |
|  | Amyloid (-) |  | 314 | 0.047 | 0.035 | 0.175 |  |  |  |  |
|  | Amyloid (+) |  | 843 | 0.096 | 0.035 | **0.007** |  |  |  |  |
|  | ADAS-cog 13 |  |  |  |  |  |  |  |  |  |
|  | All subjects |  | 1495 | -0.192 | 0.071 | **0.007** |  | -0.034 | 0.016 | **0.033** |
|  | Male |  | 850 | -0.189 | 0.087 | **0.031** |  | -0.019 | 0.020 | 0.345 |
|  | Female |  | 645 | -0.189 | 0.120 | 0.115 |  | -0.051 | 0.026 | **0.048** |
|  | ε4 non-carrier |  | 786 | -0.147 | 0.090 | 0.101 |  | -0.025 | 0.016 | 0.131 |
|  | ε4 carrier |  | 709 | -0.182 | 0.107 | 0.089 |  | -0.031 | 0.028 | 0.267 |
|  | Amyloid (-) |  | 359 | -0.100 | 0.100 | 0.316 |  | -0.020 | 0.016 | 0.220 |
|  | Amyloid (+) |  | 882 | -0.228 | 0.098 | **0.020** |  | -0.038 | 0.021 | 0.077 |
| ROS/MAP dataset | | | | | | | | | | |
|  | Global cognition score |  |  |  |  |  |  |  |  |  |
|  | All subjects |  | 1084 | 0.059 | 0.042 | 0.158 |  | 0.024 | 0.011 | **0.021** |
|  | Male |  | 361 | 0.007 | 0.078 | 0.924 |  | 0.015 | 0.018 | 0.395 |
|  | Female |  | 723 | 0.090 | 0.049 | 0.066 |  | 0.029 | 0.013 | **0.022** |
|  | ε4 non-carrier |  | 800 | 0.060 | 0.045 | 0.178 |  | 0.020 | 0.010 | 0.050 |
|  | ε4 carrier |  | 284 | 0.004 | 0.097 | 0.966 |  | 0.023 | 0.027 | 0.391 |

ADNI = Alzheimer’s Disease Neuroimaging Initiatives, CSF = cerebrospinal fluid, ADAS = Alzheimer’s disease assessment scale, ROS = Religious Orders Study, MAP = Memory and Aging Project, Aβ = amyloid beta

^†^ Multiple linear models accounting for age, sex, *APOE* genotype, and educational attainment were tested. Regression statistics of the main effect “rs72814833” in each model are shown.

^‡^ Linear mixed-effects models accounting for age, sex, *APOE* genotype, and educational attainment were tested. Regression statistics of the interaction term “rs72814833 * time” in each model are shown.
