# Supplementary material for: BMI1 is associated with CSF amyloid-β and rates of cognitive decline in Alzheimer’s disease
Source: Alzheimers Res Ther. 2021 Oct 5;13:164. doi: 10.1186/s13195-021-00906-4 (PMC8493672; doi:10.1186/s13195-021-00906-4)
Supplement: Supplementary file 4 — Additional file 4: Supplementary Table 2. Regulatory effects of the two SNPs of the BMI1 gene (HaploReg, v4.1, update 05.11.2015). Annotation results of BMI1 rs17415557 and rs72814833 from HaploReg v4.1web tool. [file 13195_2021_906_MOESM4_ESM.docx]

| Chr | POS (hg38) | LD (r2) | LD (D') | variant | Ref | Alt | AFR freq | AMR freq | ASN freq | EUR freq | SiPhy cons | Promotor histone marks | Enhancer histone marks | DNAse | Proteins bound | Motifs changed | NHGRI/EBI GWAS hits | GRASP QTL hits | Selected eQTL hits | GENCODE genes | dbSNP func annot |
| --- | --- | --- | --- | --- | --- | --- | --- | --- | --- | --- | --- | --- | --- | --- | --- | --- | --- | --- | --- | --- | --- |
| 10 | 22311698 | 1 | 1 | rs17415557 | T | G | 0.01 | 0.05 | 0 | 0.09 |  |  | BLD | BLD, BLD |  | 7 altered motifs |  |  |  | 4.3kb 5' of COMMD3 |  |
| 10 | 22320402 | 0.9 | 0.95 | rs72814833 | C | T | 0.01 | 0.04 | 0 | 0.09 |  | 23 tissues | 5 tissues | 31 tissues | EGR1 | Dmbx1 |  |  |  | BMI1 | intronic |

Supplementary Table 2. Regulatory effects of the two SNPs of the *BMI1* gene (HaploReg, v4.1, update 05.11.2015)
